# FLT3-ITD promotes immune checkpoint CD80 via ROS elevation in acute myeloid leukemia

**DOI:** 10.3389/fimmu.2025.1577313

**Published:** 2025-07-17

**Authors:** Libo Yan, Weiming Zhang, Qiyan Mo, Daogang Wang, Ning Xu, Mengzhe Yang, Tao Ren

**Affiliations:** ^1^ Xianning Medical College, Hubei University of Science and Technology, Xianning, China; ^2^ Department of Oncology, Wuming Hospital of Guangxi Medical University, Nanning, China; ^3^ Department of Clinical Medicine, The First Affiliated Hospital of Guangxi University of Chinese Medicine, Nanning, China; ^4^ Department of Clinical Medicine, The Fifth Affiliated Hospital of Guangxi Medical University, Nanning, China; ^5^ Department of Scientific Research, Beijing Friendship Hospital, Capital Medical University, Beijing, China; ^6^ Department of Oncology, The Fifth Affiliated Hospital of Guangxi Medical University, Nanning, China

**Keywords:** immune checkpoint, CD80, ROS, FLT3-ITD, leukemia

## Abstract

Acute myeloid leukemia (AML), a malignant hematological stem cell disease, arises from the malignant transformation of myeloid progenitor cells. Among the genetic aberrations in AML, mutations in the tyrosine kinase receptor FLT3, especially FLT3-ITD, are most frequently detected and are correlated with poor clinical outcomes. Intriguingly, FLT3-ITD is implicated in immune escape, although the underlying mechanism remains elusive. The present study aims to elucidate the relationship between FLT3-ITD and the immune checkpoint molecule CD80, which is crucial for immune regulation. Our results provide compelling evidence that a moderate level of CD80 localizes on the cell surface of FLT3-ITD AML cells. Mechanistically, FLT3-ITD upregulates CD80 expression by increasing intracellular reactive oxygen species (ROS) levels and subsequent CD80 enhancement. Significantly, we found that treatment with a HIF-1α inhibitor selectively suppressed the proliferation of FLT3-ITD-positive leukemic cells and induced excessive ROS production, which consequently led to CD80 overexpression. Collectively, our findings unravel the molecular pathway through which FLT3-ITD augments CD80 expression via ROS, suggesting a potential immune evasion. Moreover, this study points to a novel therapeutic strategy that combines chemotherapy-induced CD80 overexpression with immune checkpoint-targeted immunotherapy to eradicate FLT3-ITD AML cells.

## 1 Introduction

Acute myeloid leukemia (AML) represents the most prevalent form of acute leukemia in adults. Notwithstanding the remarkable progress in targeted therapies for AML, the fundamental treatment modalities have remained largely static for nearly three decades. The heterogeneity of AML is vividly demonstrated by hundreds of genomic variants (1, 2). FLT3-ITD, a constitutively active mutant variant of the receptor tyrosine kinase FLT3, is present in up to 25 - 30% of AML patients (3). The detection of the FLT3-ITD mutation at initial diagnosis correlates with an elevated risk of relapse and an unfavorable clinical prognosis, including immune escape via a presently unidentified mechanism (4).

Multiple immune evasion pathways have been discerned, and therapeutic strategies that stimulate anticancer immunity by impeding immune checkpoints have generated significant enthusiasm (5). Cancer cells frequently commandeer immune checkpoint pathways as a means of immune resistance, predominantly by expressing ligands that are recognized by inhibitory receptors on T cells. Currently, the majority of investigations concentrate on the PD-L1/PD-1 pathway (6–8). PD-L1, serves as a pivotal “don’t find me” signal for the adaptive immune system, while CD47 functions as a critical “don’t eat me” signal for the innate immune system and regulates adaptive immune responses (9). These molecules are frequently overexpressed on human tumors and therapeutic inhibition often elicits anti-tumor immune responses, a strategy that has recently been translated to clinical practice with highly promising outcomes (9). Regrettably, other immune checkpoints such as CD80 have received comparatively scant attention.

CD80, a costimulatory molecule categorized as an immune checkpoint, plays pivotal roles in the immune system. It is essential for maintaining self-tolerance and tempering immune responses to mitigate tissue damage (5). CD80 shares ligand receptors, namely CD28 and CTLA-4, on T cells (10). Notably, CTLA-4 exhibits a substantially higher affinity than CD28 (11, 12). CD28 functions as a co-stimulatory cell surface signaling molecule, while CTLA-4 serves as a co-inhibitory cell surface signaling molecule, both of which are recognized as critical for the initiation and regulation of immune responses, respectively (13). Intriguingly, moderate cell surface expression of CD80 has been reported as an immune evasion strategy in certain cancers, such as colon carcinoma and even tumor-initiating stem cells (14, 15). Nevertheless, the status of CD80 in FLT3-ITD+ AML and the underlying molecular mechanism remain enigmatic.

In the present study, we endeavor to ascertain the expression pattern of CD80 in FLT3-ITD+ AML cells and disclose the molecular underpinnings by which FLT3-ITD drives the upregulation of CD80. This investigation aims to furnish a theoretical foundation for the treatment of FLT3-ITD+ AML through the modulation of CD80 expression.

## 2 Materials and methods

### 2.1 Cells and reagents

RS4;11 and MV4–11 cell lines were procured from DSMZ and cultured in RPMI 1640 medium supplemented with 10% heat-inactivated fetal bovine serum, 100 U/mL penicillin, and 100 μg/mL streptomycin. Chemicals, including quizartinib and HIF-1α inhibitor (Selleckchem, USA), were dissolved in DMSO. Hydrogen peroxide solution was newly sourced (Sigma-Aldrich, USA). The following antibodies were employed: Mouse anti-CD80 antibody (Abcam, UK) and Goat Anti-Mouse IgG H&L (Alexa Fluor 555) (Abcam, UK).

### 2.2 Immunofluorescence staining and confocal microscopy

Whole-wheat germ agglutinin conjugated with AlexaFluor 488 was utilized to stain the plasma membrane of poly-L-lysine-immobilized cells on ice. Initially, cells were fixed with 4% formaldehyde and then blocked for 30 minutes at room temperature (RT) in PBS containing 0.5% bovine serum albumin (BSA). Subsequently, primary antibodies and fluorophore-conjugated secondary antibodies were incubated. After washing and mounting (Vector laboratories, USA), the cells were examined under a confocal microscope.

### 2.3 Flow cytometry

The expression levels of cell surface proteins were determined by flow cytometry. Briefly, non-permeabilized cells were harvested and washed. Incubation in 0.5% BSA blocking buffer was carried out to prevent non-specific staining. Primary antibodies and fluorophore-conjugated secondary antibodies were then incubated successively. Following washing, the cells were analyzed using the FACSAria flow cytometer (BD Biosciences, USA).

### 2.4 Intracellular ROS detection

ROS fluorescence signals were detected with the CellROX Oxidative Stress Reagents Kit (Thermo Scientific, USA) and analyzed by FACSAria flow cytometry following the manufacturer’s instructions.

### 2.5 Transcriptomic analyses in TCGA public database

Publicly available data were obtained from 173 AML patients from the TCGA database (https://portal.gdc.cancer.gov/), including 52 cases of the FLT3-ITD group and 121 cases of FLT3-WT group. Statistical analysis of CD80 mRNA expression was performed in R environment (v4.3.3): data were first imported using the readxl package, then cleaned and grouped with the tidyverse package. Following confirmation of non-normal distribution by Shapiro-Wilk test (P<0.05), Wilcoxon rank-sum test was used for intergroup comparison. A log1p transformation [log(x+1)] was applied to address the right-skewed distribution of expression values (ranging from 0 to 300 with major clustering around 50). Descriptive statistics (sample size, mean, median, standard deviation, and extreme values, etc.) for each group were then calculated.

### 2.6 Cell viability assay

Cell viability was evaluated using the CellTiter-Glo Luminescent Assay (Promega, USA) as per the manufacturer’s guidelines, with readings obtained using a multilabel plate reader (Perkin Elmer, USA). The results of tested chemical effects were normalized against the parallel DMSO controls.

### 2.7 Statistics

All experimental data were presented as the mean ± standard error of the mean (SEM). Statistical significance between two groups was assessed using the Student’s t-test. A p-value less than 0.05 was deemed significant.

## 3 Results

### 3.1 Moderate expression of immune checkpoint CD80 on the cell surface of FLT3-ITD+ AML

**Figure 1:**
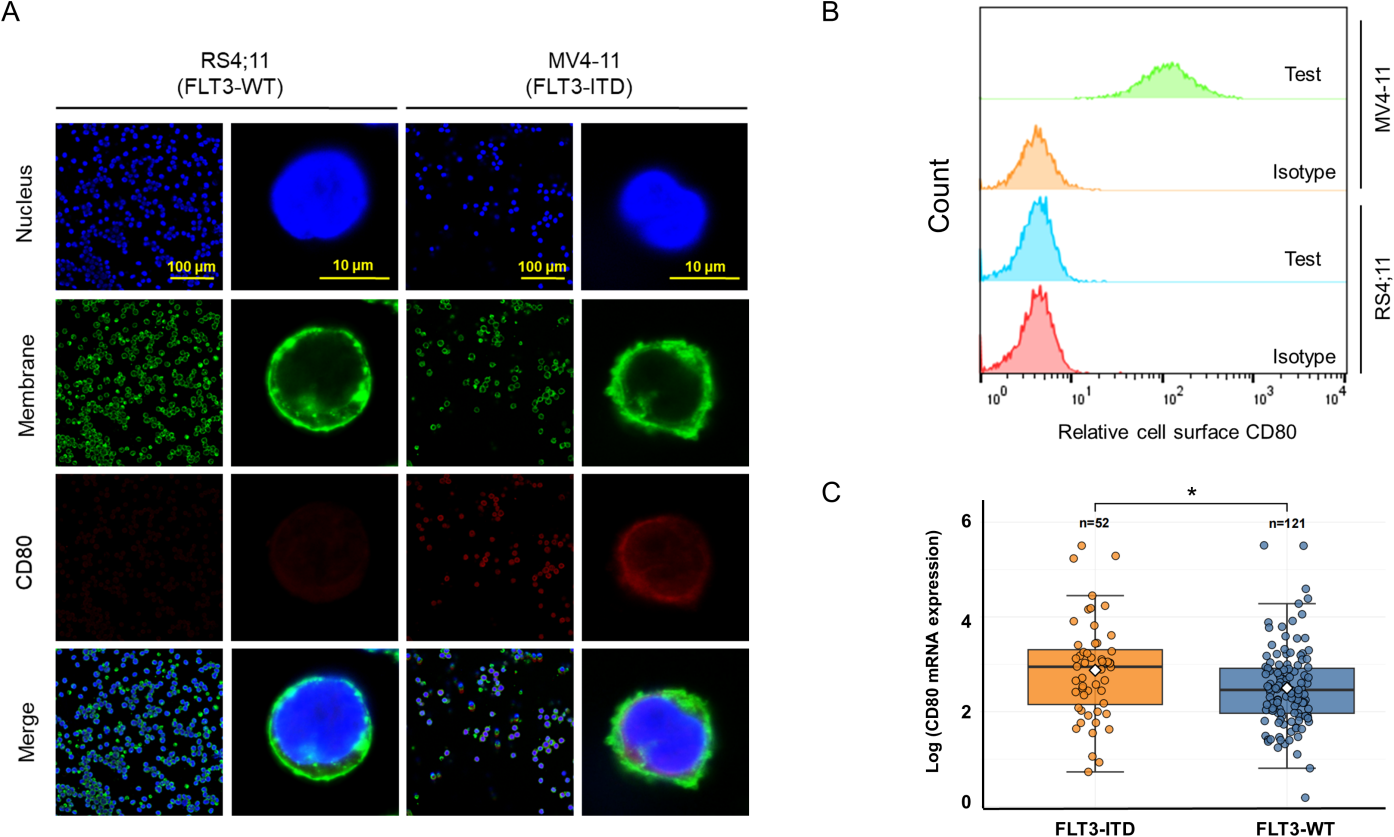
Moderate expression of CD80 on the surface of FLT3-ITD AML cells. **(A)** Immunofluorescence staining and microscopy observation for the expression and localization of CD80 in RS4;11 cells (harboring FLT3-WT) and MV4–11 cells (exclusively FLT3-ITD). **(B)** Flow cytometry analyses of cell surface CD80 in RS4;11 and MV4–11 cells. **(C)** The transcriptomic analyses in patient-derived AML samples in TCGA public datasets including these FLT3-ITD AML patients and the FLT3-WT AML patients. * indicating p <0.05.

To explore the immune checkpoint CD80 status on leukemic cell surface, immunofluorescence staining was employed to investigate its expression of RS4;11 cells (FLT3-WT) and MV4–11 cells (harboring FLT3-ITD). Our findings revealed that CD80 was predominantly expressed on the cell surface (**Figure 1A**). Notably, in FLT3-ITD+ MV4–11 cells, CD80 exhibited a moderate level of expression, whereas its expression was relatively faint in the wild-type FLT3-expressing RS4;11 cells. To further quantification of CD80 expression, we conducted the flow cytometry analyses of cell surface CD80 and the results indicated that FLT3-ITD AML cells (MV4-11) displayed remarkable CD80 cell surface expression, but not the FLT3-WT leukemic cells (RS4;11) (**Figure 1B**). In addition, we also explored transcriptomic analyses using patient-derived AML samples in TCGA public datasets and discovered the mRNA level of CD80 was significantly higher in these FLT3-ITD AML patients compared with the FLT3-WT AML patients (**Figure 1C**).

**Figure 2:**
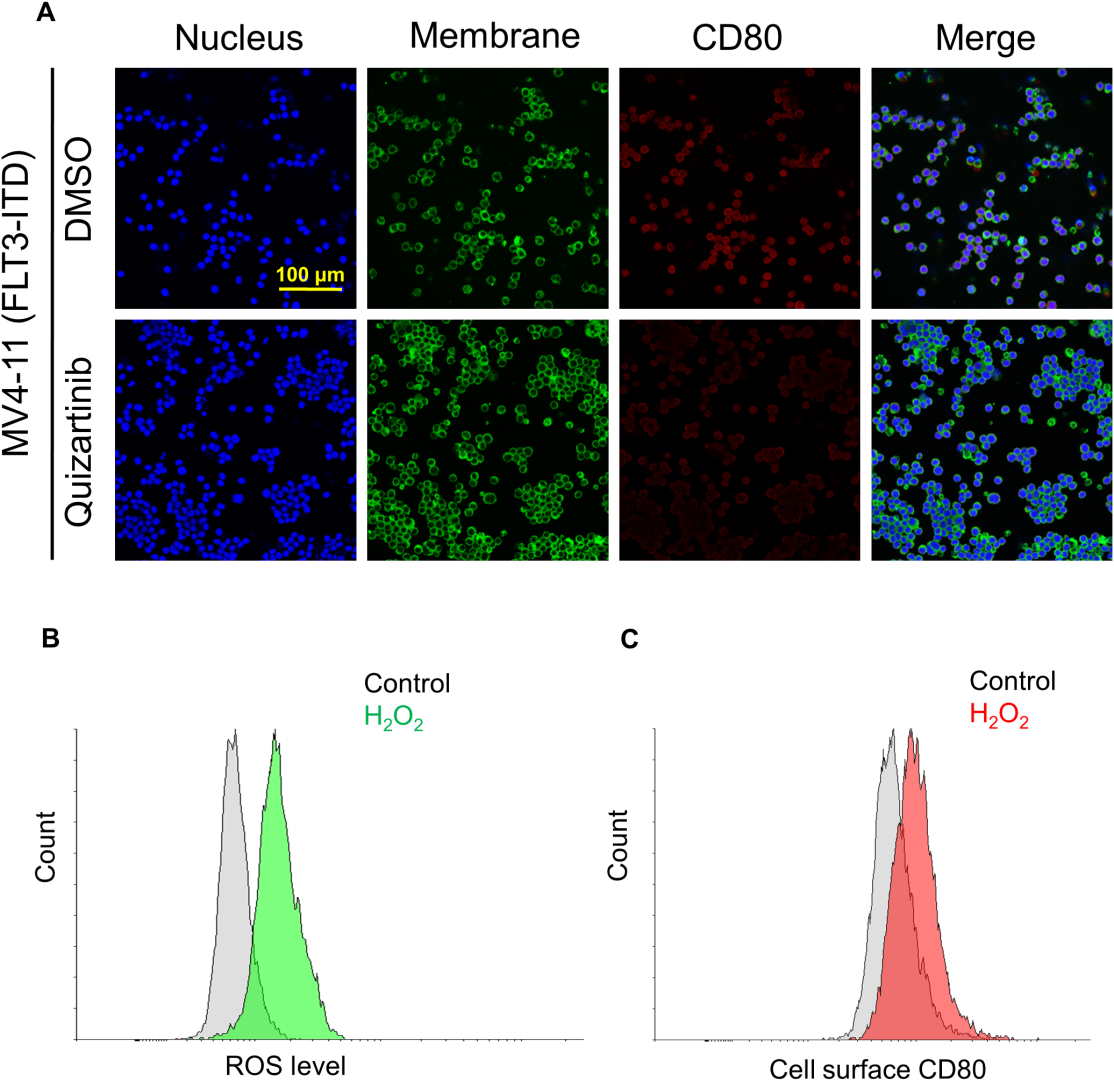
FLT3-ITD mutation promotes CD80 expression via activated receptor tyrosine kinase and associated with intracellular ROS level. **(A)** Cell surface CD80 expression in MV4–11 cells without or with treatment of tyrosine kinase inhibitor (TKI) quizartinib at a concentration of 100 nM for 24 h. **(B)** Flow cytometry analyses of the ROS levels in MV4–11 cells with 0.1% H^2^O^2^ for 24 h. **(C)** Flow cytometry analyses of the cell surface CD80 expression in MV4–11 cells under the same treatment conditions (0.1% H^2^O^2^ for 24 h).

To further validate the role of FLT3-ITD in promoting CD80 expression and elucidate the relationship between FLT3 tyrosine kinase activity and CD80 expression, MV4–11 cells were treated with quizartinib, a tyrosine kinase inhibitor (TKI). Immunofluorescence staining demonstrated an obvious reduction in CD80 expression following quizartinib treatment (**Figure 2A**).

Collectively, these experimental results indicate that immune checkpoint CD80 predominantly localized on the cell surface is moderately expressed in FLT3-ITD+ MV4–11 cells and correlated with the tyrosine kinase activity.

### 3.2 FLT3-ITD mutation promotes CD80 expression via elevated intracellular ROS level

Reactive oxygen species (ROS) play crucial roles in the immune system (16). Based on this, we hypothesized that the elevated ROS level, as a consequence of the constitutive activation of FLT3-ITD signaling cascades (17), might act as a central regulator in driving CD80 expression. To test this hypothesis, MV4–11 cells were firstly treated with H^2^O^2^ to mimic the cellular hypoxia microenvironment. The results showed a marked increase in cell surface CD80 expression concomitant with a surge in ROS production (**Figures 2B, C**). These findings jointly confirm that CD80 expression is driven by FLT3-ITD in association with elevated intracellular ROS levels.

### 3.3 HIF-1α inhibition selectively suppresses cell proliferation and augments CD80 expression through ROS overproduction

**Figure 3:**
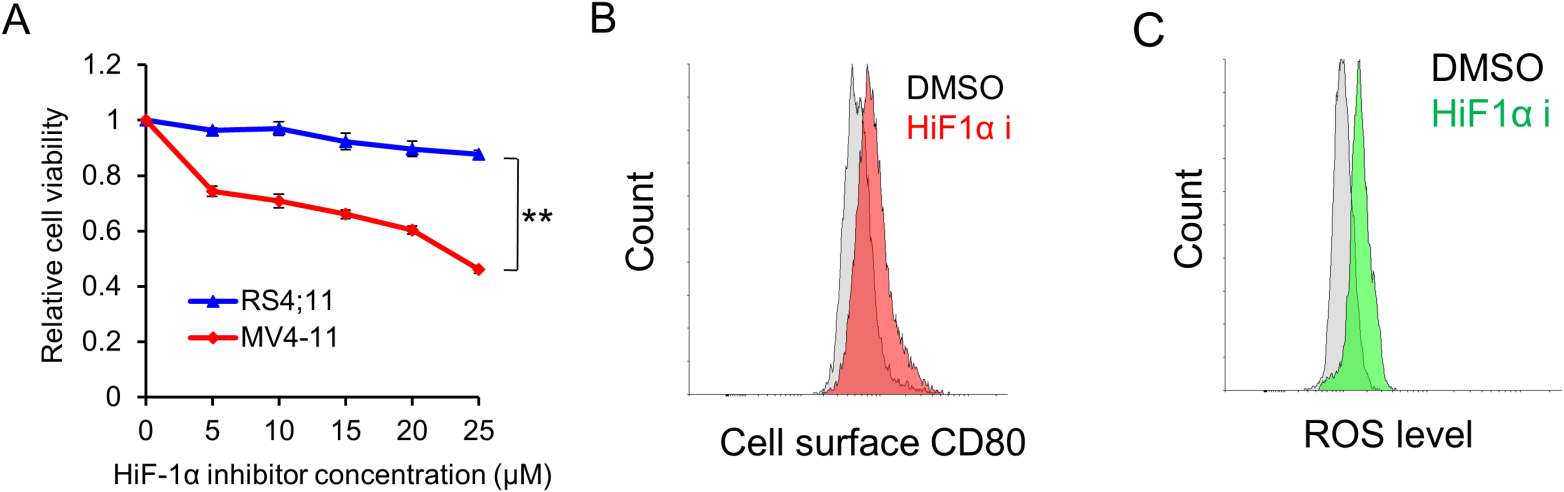
HIF-1α inhibitor selectively suppresses leukemic cell proliferation and induces higher CD80 expression via excessive ROS production. **(A)** The selective inhibitory effect of the HIF-1α inhibitor on the proliferation of FLT3-ITD-expressing cells. Cells were treated with varying concentrations of the HIF-1α inhibitor for 48 h. Subsequently, cell viability was measured using the cell titer-glo assay. ** indicating p < 0.01. **(B)** Flow cytometry analyses of the cell surface expression of CD80 in MV4–11 cells treated with vehicle (DMSO) or HIF-1α inhibitor (20 μM) for 48 h. **(C)** Flow cytometry analyses of the ROS levels in MV4–11 cells following treatment with vehicle (DMSO) or HIF-1α inhibitor (20 μM) for 48 h.

To gain a deeper understanding of the underlying mechanism and explore potential therapeutic avenues, we opted to treat these FLT3-ITD leukemic cells with a HIF-1α inhibitor, given previous reports indicating that HIF-1α drives pro-survival signaling pathways in AML (18, 19). Interestingly, we observed that the HIF-1α inhibitor markedly inhibited the proliferation of FLT3-ITD+ MV4–11 cells, while having no such significant effect on the wild-type FLT3-expressing RS4;11 cells (**Figure 3A**). Moreover, cell surface CD80 levels were found to increase following HIF-1α inhibitor treatment in MV4–11 cells (**Figure 3B**). Since HIF-1α is responsive to hypoxia stress, we further examined the ROS level in MV4–11 cells and found that it increased after HIF-1α inhibitor treatment (**Figure 3C**).

Taken together, these data provided insights into the relationship between HIF-1α inhibition, ROS production and CD80 expression, which suggested that FLT3-ITD maintains a moderate ROS level, serving as a pro-survival signal and drives a moderate amount of CD80 expression. Upon HIF-1α inhibition, further ROS overproduction functions as a pro-death signal and induces CD80 overexpression.

## 4 Discussion

In the current study, we made the notable discovery that the immune checkpoint CD80 was moderately expressed in FLT3-ITD AML cells, in contrast to its faint expression in wild-type FLT3 leukemic cells. To rule out the potential confounding factor of genetic background differences among cell lines and firmly establish the relationship between FLT3-ITD and CD80, we treated FLT3-ITD positive leukemic cells with the tyrosine kinase inhibitor (TKI), quizartinib. Our results unequivocally demonstrated that CD80 expression was significantly diminished following TKI treatments.

Furthermore, we observed that the HIF-1α inhibitor selectively impeded the proliferation of FLT3-ITD positive leukemic cells. This was consistent with the previous study that Wang et al. had previously reported that targeting HIF-1α could eradicate cancer stem cells in hematological malignancies (18, 19). However, our novel finding was that HIF-1α inhibition led to CD80 overexpression in FLT3-ITD positive leukemic cells, concurrent with a surge in ROS production. This CD80 overexpression phenotype could be replicated by treating cells with hydrogen peroxide solution, which induced a more pronounced hypoxia microenvironment through excessive ROS generation.

The highlights in current study include: A moderate quantity of immune checkpoint CD80 is expressed on the cell surface of FLT3-ITD-positive acute myeloid leukemia; FLT3-ITD augments CD80 expression via an elevated intracellular level of reactive oxygen species (ROS); The HIF-1α inhibitor selectively suppresses the proliferation of FLT3-ITD-positive leukemic cells and triggers CD80 overexpression as a result of excessive ROS generation. Consistent with the cell line models, the transcriptomic analyses using patient-derived AML samples in TCGA datasets indicated that the mRNA level of CD80 was significantly higher in these FLT3-ITD AML patients compared with the FLT3-WT AML patients. The collections of more clinical samples will be performed to further explored the associated mechanisms in future. The oncogenic FLT3-ITD triggers ligand-independent receptor dimerization and activation, subsequently leading to the constitutive activation of downstream signaling cascades, such as PI3K/AKT, STATs, and MAPKs, promoting cell differentiation, proliferation, and survival (20). Pathologically, the FLT3-ITD mutation itself can elevate endogenous ROS levels in leukemic cells via multiple pathways, including STAT5 (17, 21), which likely accounts for the moderate CD80 expression in FLT3-ITD AML cells. Collectively, our data suggest that ROS could serve as a central mediator in the adaptive response of cancer cells to hypoxia, particularly in relation to immune checkpoints in FLT3-ITD leukemic cells. Undoubtedly, our study provides valuable insights into the mechanisms of action of chemotherapeutics that increase ROS levels. While a moderate ROS level can fuel cancer progression, it also renders cancer cells more susceptible to additional stresses (22–24). Strategically targeting ROS levels could potentially undermine cancer cell survival (25).

**Figure 4:**
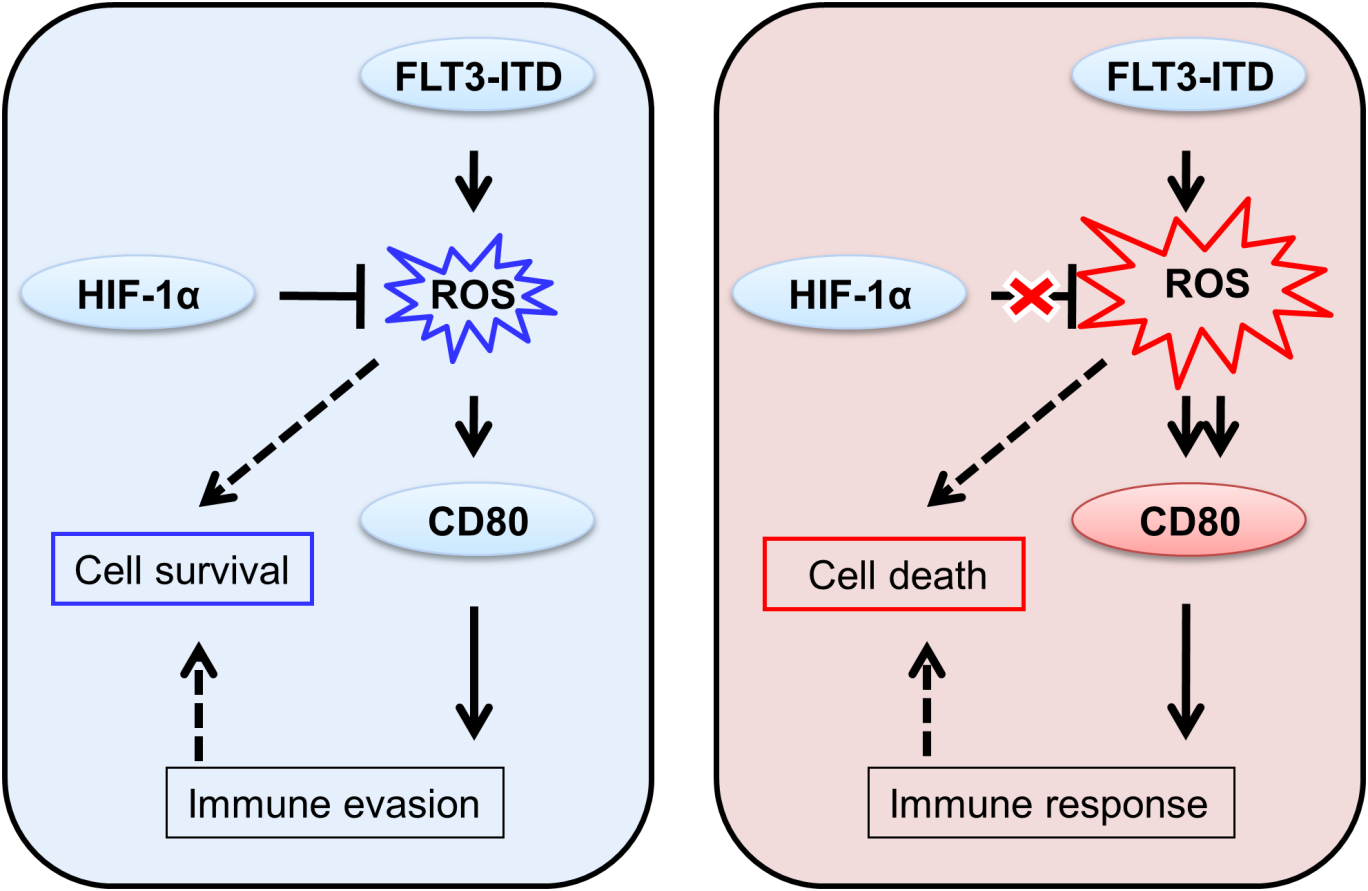
Hypothesis of the differential immune responses to moderate and overexpressed CD80 proteins in FLT3-ITD+ leukemic cells.

Herein, we postulated that the elevated ROS level, as an added stressor, could not only induce cancer cell death through direct cytotoxicity but also augment the adaptive immune response when combined with other therapies such as chemical drugs. We proposed that, in AML, FLT3-ITD mutation often produce ROS for cell survival regulated by HIF-1α, which also induce CD80 for the potential immune invasion. However, when the balance of HIF-1α regulation was disturbed, overwhelming ROS was produce caused cell death and enhanced CD80 might also trigger immune response (**Figure 4**). During the immune response, T cells undergo a progressive differentiation from naive to effector cells. The co-stimulatory molecule CD28 is predominantly expressed on the surface of naive T cells, whereas the co-inhibitory molecule CTLA-4 is mainly found on effector T cells (13). The moderate levels of CD80 proteins on FLT3-ITD positive leukemic cells might preferentially bind to CTLA-4 rather than CD28, due to their differential affinities. This CD80/CTLA-4 interaction could potentially exhaust effector cells, while naive cells would fail to execute effector functions in the absence of sufficient CD80/CD28 stimulatory signaling. Indeed, Kamphorst et al. recently reported that PD-1/PD-L1 cancer immunotherapy also necessitates CD28 co-stimulation for CD8 T cell rescue, highlighting the crucial role of the CD80/CD28 pathway (26). Numerous chemotherapeutic drugs and irradiation have been reported to modulate ROS levels in cancer cells and upregulate immune checkpoints like CD80, corroborating our current findings (27, 28). Moreover, transfection of CD80 plasmids into colon carcinoma cells enhanced immunogenicity and led to tumor rejection, whereas silencing CD80 abrogated tumorigenicity (14). Additionally, similar to the overexpression of the co-stimulatory molecule CD80 in cancer cells, a soluble form of CD80 has also been demonstrated to potentiate antitumor immunity (29). As for therapeutic applications, our study indicated that FLT3-ITD promotes immune checkpoint CD80 via ROS elevation in AML and the HIF-1α inhibition, further ROS overproduction functions as a pro-death signal and induces CD80 overexpression. In the future, the combined therapies might benefit the patient outcome based on the present basic research rational. Furthermore, our current study indicates that HIF-1α inhibition exhibits selective targeting of FLT3-ITD+ leukemic cells, offering critical insights for future clinical drug development. This finding didn’t highlight potential off-target effects and the therapeutic synergies with existing AML treatments, warranting further investigation for translational applications. Taken together, these studies potentially unveil an additional immunotherapeutic mechanism underlying these anticancer modalities in clinical practice.

In conclusion, our study has elucidated the expression profile of CD80 in FLT3-ITD AML cells and proposed a hitherto unrecognized hypothesis that ROS drives CD80 expression as a regulator of the cancer cell adaptive response. This discovery points to a novel avenue for immunotherapy, in combination with other anticancer treatments, which could be harnessed to target CD80 expression levels and ultimately eradicate FLT3-ITD AML cells in future.

## Data Availability

The original contributions presented in the study are included in the article/Supplementary Material. Further inquiries can be directed to the corresponding authors.
